# The psychosocial impact of climate change among smallholder farmers: a potential threat to sustainable development

**DOI:** 10.3389/fpsyg.2023.1067879

**Published:** 2023-04-26

**Authors:** Dumisani Shoko Kori

**Affiliations:** Department of Geography, Environmental Management and Energy Studies, Faculty of Science, University of Johannesburg, Johannesburg, South Africa

**Keywords:** climate change, emerging rural communities, environmental psychology, land reform, smallholder farmers, social wellbeing, sustainable development goals

## Abstract

Psychosocial impacts of climate change and implications on sustainable development remain unclear. This problem was addressed focusing on smallholder farmers in resettlement areas of Chirumanzu District, Zimbabwe. An Exploratory Descriptive Qualitative research design was adopted. Purposive sampling techniques were used to select 54 farmers who served as main respondents from four representative wards. Data were collected through semi-structured interviews and analyzed using a grounded theory approach. Code groups and codes were established through inductive approaches considering narratives of farmers. Forty psychosocial impacts were established. They were qualitative, intangible, indirect and difficult to measure quantitatively. Farmers agonized over the threat of climate change on farming operations, felt humiliated, and embarrassed over detestable practices they resorted to due to climate change. Some farmers experienced heightened negative feelings, thoughts, and emotions. It was established that psychosocial impacts of climate change have a bearing on sustainable development of emerging rural communities.

## 1. Introduction

### 1.1. Background

Climate change is not just an environmental, but a psychological (Clayton, 2020) and social problem as well. Similarly, climate change does not only affect physical human health, but also mental health (Dodgen et al., 2016; Manning and Clayton, 2018), and social wellbeing. Although research has been conducted on the impacts of climate change on physical and mental health as well as social aspects associated with climate change, not much has been done on smallholder farmers in developing countries who take farming as their main source of livelihood. Much of the research that illustrates climate change adversities on smallholder farmers have been conducted in the developed countries. Charlson et al. (2021) conducted a scoping review to assess the body of literature on climate change and mental health and found that, out of a sample of 120 studies conducted between 2001 and 2020, most studies (87) were conducted in high income countries with Australia having the most research studies (34), followed by Canada and the United States of America (USA), (17 and 16, respectively). The assessment also revealed that only 3% of the studies were conducted in low-income countries.

Among the studies conducted in Australia is an early study by Hanigan et al. (2012), who investigated the relationship between suicide and drought among male farmers in South Wales, Australia. It was found that an increase in drought index increased the relative risk of suicide by 15% in rural males in the age range, 30–49 years. Ellis and Albrecht (2017) also explored the significance of sense of place in understanding the mental health impacts of climate change on farmers in Western Australia Wheatbelt. One of the findings was that climate change increased farmers’ worry about weather patterns, disregarded notions of self-identity, and led to place-based distresses. Fuentes et al. (2020) examined environmental changes’ impacts on wellbeing in selected indigenous communities in the boreal forest of eastern Canada. The study showed that the felt impacts were more for participants with higher quality of life. In the United States, Howard et al. (2020) sought to understand farmer and rancher perception toward climate change impacts on mental wellbeing in rural areas along the Western parts of the country. It was observed that there was a moderate correlation between climate risk perception and anxiety related to climate impacts.

In support with the observations made by Charlson et al., 2021, that studies on the impacts of climate change on mental health in developing countries are limited. Atwoli et al. (2022) reviewed evidence of climate change impacts on mental health in Africa. The review demonstrated that studies in the research area in Africa are scarce and recommended that national governments should seriously consider it as an emerging threat to the region. Among the few assessments focusing on the impact of climate change on mental health of smallholder farmers in developing countries, is a study by Acharibasam and Anuga (2018) who sought to understand and explain the association between climate change and farmers’ emotional regulation practices among smallholder farmers in Northern Ghana. The study concluded that the impact of climate change on farmers’ emotional regulation practices was likely to predispose them to mental health problems later in their lives. This imbalance in research on impacts of climate change and mental health between the developed and developing countries projects a gloomy picture because impacts on the individual level across the world are significant considerations for the Intergovernmental Panel on Climate Change (IPCC, 2022) and climate change science (Reser et al., 2012).

It is also noticeable that the link between the psychosocial impacts of climate change and sustainable development are not clear, if not missing. A few studies attempt to determine climate change impacts on sustainable development issues (Reyer et al., 2017), especially in emerging rural communities. The scant literature on the psychological impacts of climate change, for example (Grothmann and Patt, 2005; Fritze et al., 2008) forms a solid base on the subject. However, it does not incorporate the social impacts dimension that may have a bearing on the psychological effects experienced. The social impacts of climate change are defined as the negative effects that diminishes the wellbeing of people resulting in increased poverty, food insecurity, and gender inequality with the effect of reducing the quality of life of individuals and communities (Safonov, 2019). Doherty and Clayton (2011) defined psychological impacts of climate change as the direct and indirect effects of climatic changes. The direct effects are deemed “acute or traumatic effects of extreme weather events” while the indirect are considered threats to emotional wellbeing due to concern and uncertainty of future risks. This study adopted some of the elements provided in the definitions above. Therefore, for the purpose of the study, the psychosocial impact of climate change is the influence of the social impact on the mental wellbeing of smallholder farmers. There is a connection between the social and psychological impact of climate change in that the former generate the later. The term psychosocial is used to illustrate this connection. However, it should be noted that although the social and psychological impacts are connected, they were separated for ease of analysis.

Social and psychological impacts of climate change are rarely analyzed together in existing literature. For example, Njeru et al. (2022) investigated the effect of climate change on mental health among smallholder crop farmers in Kenya but did not incorporate the social aspects. The scant literature that attempt to intergrate the social and psychological impacts of climate change do not show the implications on sustainable development of emerging rural communities. For example, Truelove et al. (2015) sought to understand the social and psychological factors that influence individual adaptation using a risk, coping, and social appraisal model; however, the study does not show any implications on sustainable development.

Climate change is one of the largest threats to individual smallholder farmers. Changes in climate that have more bearing on individual smallholder farming manifest as higher than normal temperatures, altered patterns of precipitation and intensity, and increased frequency of extreme events such as droughts and floods (Field and Barros, 2014). This trend has been observed in Zimbabwe (Mushore et al., 2021; Nciizah et al., 2021). Rainfall seasons have become unpredictable, erratic, and volatile and varying with long dry spells compounding to shorter and drier growing seasons (Chikodzi and Mutowo, 2012). The same authors also observed that droughts and floods are increasing. Shortened growing seasons, diminished water resources, and loss of agro-biodiversity systems have been cited as part of the evidence for increased climate change and variability impacts in Zimbabwe (Mapfumo et al., 2016).

Despite an increasing number in climate impact studies in Zimbabwe, literature on the psychosocial impacts of climate change is lagging. Senda et al. (2020) modeled the impacts of climate change on the productivity of rangeland and livestock populations in Nkayi District and found that low rainfall reduced herbaceous biomass production while increases in carbon dioxide concentrations in the atmosphere increased the growth of trees and shrubs. It was recommended that smallholder farmers should consider keeping livestock species that can utilize trees and shrubs. Muringai et al. (2022) assessed the impacts of climate change on small-scale fishers and showed that it was believed that increases in temperature and declining rainfall has led to reduced fish stocks and catches in the Binga and Sanyati basins. Tui et al. (2021) applied a multi-modeling approach to co-develop climate change impact scenarios under dryland framing system in Nkayi where maize-cattle systems are dominant. Reduction in maize yield due to high temperatures were observed while milk production was affected due to low production in crop residue and reduced rangeland productivity that affected cattle fodder intake. An analysis of climate change impacts on traditional farming systems in Gwanda, Mangwe, and Matobo Districts (Ndlovu et al., 2020) established that traditional farming systems were being abandoned for other livelihood options due declining annual rainfall, destructive impacts of Cyclone Eline and Dineo in 2000 and 2017, respectively, on farming infrastructure that support traditional farming ultimately leading to worsened food insecurity. A spatial model of the effects of climate change on the distribution of *Lantana camara* in Matebeleland South (Ncube et al., 2020). It is expected that the area invaded by *Lantana camara* would increase by 5,892 km^2^. Although these studies are of great importance and forms a basis for climate change adaptation interventions, what is missing from literature is the psychosocial impact of climate change on smallholder farmers and their implications on sustainable development. Focus is largely on the impact of climate change on plant growth, yield and productivity, livelihoods and ecosystem balance, and sustainability.

The problem stated in the preceding paragraphs is perpertuated by several factors. First, the bias toward quantitative measures of the impact on crop growth and yield worsen this challenge. The fact that social and psychological impacts are difficult to measure could also be contributing to this challenge. Second, scope is important when analyzing climate change impacts. The scant research that tackles psychological impacts were done in the developed world and rarely are individual smallholder farmers in developing countries like Zimbabwe included. Third, the impacts of climate change impacts on sustainable development are highly dynamic and difficult to project (Reyer et al., 2017). It seems this is one of the reasons why many researchers are not very keen to explore this dimension. Furthermore, there is little knowledge based on psychosocial impacts associated with climate change especially at individual farm level (Ghimire and Huang, 2016). These reasons could explain why the full impacts of climate change are underestimated as postulated by Thornton et al. (2014). This may be extended to underestimates of psychosocial impacts of climate change to sustainable development initiatives. Consequently, there is a dearth of knowledge that value the scourge, agony, and trauma inflicted on farmers by climate change and associated implications on sustainable development.

Clayton (2020) recommended that the social consequences of climate change also need to be addressed in order to get a complete picture of the psychological experiences. This study was conceived with this recommendation in mind and aimed to establish the implications of the psychosocial impacts of climate change on sustainable development among smallholder farmers in Chirumanzu. This is also in response to a call by Reyer et al. (2017) that studies to establish linkages between climate impacts and development are needed to gain a clearer picture of the relationship. To update and extend literature on the subject, this study explored the psychosocial impacts of climate change and implications on sustainable development on smallholder farmers in resettlement areas of Chirumanzu District of Zimbabwe.

### 1.2. Study context and research questions

Most pre-colonized states in Africa have shifted their focus toward land and agrarian reforms as a development initiative. Zimbabwe adopted a land reform program from 1980 after independence, which was done in two phases, the old resettlement of 1980–1999 and the Fast Track Land Reform Program (FTLRP) of 2000. The land reform program resettled people under Model A1 and A2. This research focused on beneficiaries of the A1 Model. Model A1 was designed to address poverty and vulnerability for the landless poor and to decongest overpopulated communal areas (United Nations Development Program, 2002). As a result, farmers in Model A1 resettlement program were allocated small plots to grow crops and for animal grazing (Mkodzongi and Lawrence, 2019). Model A1 has three settlement schemes, old resettlement, villagised and self-contained (Njaya and Mazuru, 2014). In the old resettlement and A1 villagised scheme, each farmer is allocated about 1 ha of land to build homesteads making up villages. Pieces of land ranging from 5 to 6 ha are located in a different area away from the homesteads and farmers share a common grazing area. In self-contained scheme, plots between 15 and 30 ha are allocated per individual and are used for both cultivation and grazing.

Resettlement farmers in Zimbabwe including the Model A1 farmers exist under pronounced political and economic marginalization as the state restrict entry of external actors into these areas (Chiweshe, 2014). Civil society organizations and international donors are not interested in working in resettlement areas, which they regard to be “contested lands.” Scientific researchers shun resettlement areas in favor of communal lands. This is evidenced by an imbalance in research carried out in the communal areas compared to resettlement areas. Although the Government of Zimbabwe (2015) acknowledged that farmers in resettlement areas are vulnerable to climate change, it does not have a clear roadmap nor resources to support them and this compounds the situation. To date, resettled farmers remain isolated from external funding, resource aid and research enquiry. These facts triggered the need for a study designed to investigate the psychosocial impacts of climate change on smallholder farmers in Chirumanzu District of Zimbabwe and implications on sustainable development in this emerging rural community.

This study intends to build literature that would be infused into the existing bio-physical climate impacts that are vast and extensive. The major questions underpinning this study are: what are the social and psychological impacts of climate change on smallholder farmers in resettlement areas of Zimbabwe? What are the implications of social and psychological impacts of climate change on sustainable development? The study draws upon selected Sustainable Development Goals (SDGs) of the United Nations (UN) (2015) to articulate the implications of psychosocial impacts of climate change on sustainable development. There is consensus among scientists and policy makers that climate change is happening and that appropriate action is required to mitigate and adapt to the effects. This study is important for advancing our understanding of psychosocial processes that underlie sustained engagement in pro-environmental behaviors that enhance sustainable development.

## 2. Materials and methods

This study utilizes data collected for PhD research titled, “developing a framework for estimating adaptation cost to climate variability and change”. The study was a case study of smallholder maize farmers in resettlement areas of Chirumanzu District, Zimbabwe. From the PhD research, there are two journal articles and one book chapter that have been published (Kori et al., 2020, 2021; Shoko Kori and Kori, 2022). As such, there may be overlaps in terms of the description of the study area, research design, sampling, data collection, and analysis methods. However, it should be noted that the publications have different foci. The journal article, Kori et al. (2020) focuses on adaptation measures that were adopted by maize farmers to reduce the threat and impact of climate variability on maize farming. The book chapter Kori et al. (2021) focuses on the intangible and indirect costs of the adaptation measures adopted. The journal article (Shoko Kori and Kori, 2022) for focuses on the tool for estimating adaptation costs. This paper on the other hand, focuses on the implications of the psychosocial impacts of climate change on sustainable development drawing upon the SDGs for emerging rural communities like the resettled farms in Chirumanzu District.

### 2.1. Study area

Chirumanzu District is located in the Midlands Province of Zimbabwe. More than 90% of the District lies in Natural Region III with the remainder displaying characteristics of Natural Region IV (Gwamuri, 2012). Natural Regions III and IV are two of Zimbabwe’s five Natural Regions that represent agricultural potential for the production of crops and livestock. Natural Region III is a semi-intensive farming region, suitable for livestock production based on fodder crops. Natural Region IV is a semi-extensive region suitable for livestock under resistant fodder crops. Despite this, smallholder agriculture in both regions is more inclined to crop production.

Low rainfall ranging from 500 to 750 mm and 400 to 510 mm *per annum* characterize Natural Regions III and IV, respectively (Musara et al., 2011). Extreme weather events such as severe mid-season dry spells and frequent seasonal droughts are experienced (Simba and Chayangira, 2017). Despite this, rain fed agriculture is the major source of livelihoods in the area. Its rural setting within the context of resettlement and continuous battering by extreme weather events presented a suitable platform for scientific investigation. Out of the 23 wards constituting Chirumanzu District, nine are resettlement areas.

### 2.2. Research design and sampling procedure

An Exploratory Descriptive Qualitative (EDQ) research design (Hunter et al., 2019) was adopted. Exploratory Descriptive Qualitative design is a combination of the exploratory and the descriptive qualitative research designs. The exploratory design allowed the researcher to uncover the little understanding of a phenomenon as articulated by Polit and Beck (2008). In this case, the exploratory design enabled the researcher to uncover and understand the psychosocial impact of climate change on smallholder farmers and how it links to sustainable development. The qualitative design allows participants to contribute to knowledge development (Reid-Searl et al., 2012). In this study, smallholder farmers had the opportunity to take part and contribute to the development of knowledge about the psychosocial impact of climate change. The approach facilitates a more transparent and sustainable way toward development of local communities.

A two-stage sampling procedure was used. First, heterogeneous purposive sampling also known as maximum variation purposive sampling (Etikan et al., 2016), was used to select wards in which the research was carried out. Wards 11, 12, 15, and 20 were purposively sampled out of the nine wards falling under the resettlement areas in Chirumanzu. Ward 11 represented the old resettlement scheme. Ward 12 was selected to represent the villagised scheme. Ward 15 was selected to represent self-contained scheme. Ward 20 was unique because it has both the A1 villagised and self-contained resettlement schemes.

Homogenous purposive sampling (Etikan et al., 2016) was used to select A1 farmers from the previously sampled wards so that they could serve as the main respondents in the semi-structured interviews. The inclusion and/or exclusion criterion was A1 farmers who had encountered impacts of climate change. Intensive consultation with the District Agricultural Extension Officer and Ward Extension Officers led to the identification of A1 farmers who met the inclusion/exclusion criterion.

### 2.3. Ethical considerations

Approval to carry out the research was sought from the University of Venda‘s Research Ethics Committee where an Ethical Clearance Certificate was issued. Permission and approval to conduct the research was also sought from the District Administrator who granted the permission to conduct research. Informed consent was sought from the respondents through communicating the purpose of the research, how the interviews will be conducted and how the information was going to be used. This was done through communicating to the respondents the aims and purpose of the study and its implications. It was also highlighted that the respondents were under no obligation to answer any of the questions. The respondents were assured that their participation would not predispose them to any forms of harm or danger. Respondents were also informed that they have the right to withdraw at any time. A register was signed prior to the interviews, which also reflected that they had understood what was communicated to them. Privacy and confidentiality was maintained by using pseudo names, the researcher discretely guarded the register.

### 2.4. Data collection and analysis

Semi-structured interviews were conducted with A1 farmers selected. Interviews were conducted in Shona, the local vernacular language to ensure a common understanding of the questions. An interview guide was the main data collection tool used to solicit information on the social and psychological impacts of climate change on farming. The interview guide contained open-ended questions to allow farmers to elaborate more on their experiences to provide a clearer understanding of the social and psychological impacts of climate change on farming. To gather information on the social impact, farmers were asked how climate change affected farming as their main source of livelihood and what were the consequences associated with the impacts on overall harvest, surplus harvest for sale, food availability in the household, general standard of living, and general social wellbeing. To gather information on the psychological impact, farmers were asked what feelings, thoughts, and emotions were generated by the impact of climate on farming as their main source of livelihood.

Detailed notes of the interviews were taken. Concurrent audio recording of the interview proceedings helped enhance accuracy of farmers’ responses. Data saturation was reached at the eighth farmer in all wards. However, interviews were continued until the 15th farmer in Wards 11, 12, and 20. This was done in line with the advice Peterson (2019) that seeks to obtain deeper insights. However, in Ward 15, interviews were terminated after interviewing the ninth farmer due to an unexpected commotion that erupted during interviewing because unlike in other wards, interviews in Ward 15 were held at an open space and other farmers not selected to participate in the interviews also wanted to be interviewed. This created hostile conditions that made it impossible to continue with the interviews up to the 15th farmer as originally planned.

In total, 54 farmers were interviewed. A large proportion of the farmers (82%) who participated in the study were male. Farmers’ ages were skewed toward the older age groups, with 43 farmers in the 61–70 and 71–80 age groups while only two farmers were in the 31–40 age group. Farming experience varied widely from six to more than 30 years. Thirty-one farmers attained secondary education. Only three farmers had tertiary qualifications. Five farmers did not have any formal education but could read and write. Resettlement and farm details varied across the four wards and from farmer to farmer. Most of the farmers were settled during the period 1998 and 2002 reflecting that they were settled under the FTLRP. All farmers in Ward 11 were settled in the 1980s reflecting that they were settled under the old resettlement scheme.

Audio-recorded interviews were transcribed verbatim. Textual data from audio recordings and notes taken were stored as a MS Excel spreadsheet on a case-based entry as illustrated by Friese (2016). The file was saved in the “CSV Comma Delimited format” (Friese, 2016), and imported into Atlas.ti Version 8. A grounded theory approach was adopted.

Textual responses were used to develop preliminary codes through inductive coding. It was performed via open coding (Glaser, 2016) and *in vivo* coding (Manning, 2017). Open coding involved reading the text responses, sentence by sentence while forming detailed and structured themes. In this way, a grounded analysis was guaranteed. Simultaneously, codes and resulting code groups that were drawn from primary data to avoid missing important information. The same approach was used for *in-vivo* coding. In this case, a word or phrase from textual responses was used to represent a code or code group.

Similar codes were merged to avoid repetition. Irrelevant codes were deleted. Preliminary codes were grouped and merged into code groups. Groups with preliminary codes that were combined yet reflecting two or more concepts were split. Selective coding was used to create qualitative visual representations of the data in the form of network diagrams. Relationships and patterns were created using the resulting codes and groups linking them with quotations to create network diagrams, which were then exported to MS Word for use in presenting results.

### 2.5. Trustworthiness of research

Trustworthiness in the study was built through establishing credibility, transferability, dependability, and confirmability (Lincoln and Guba, 1985). To improve credibility irregular and/or contrary cases that were unique to some or one respondent were included in the analysis in line with Burnard et al. (2008) approach. Transferability was improved through establishing inferential generalization (Lewis et al., 2003). The study was carried out over a single case factoring in different soil types in the research design. The soil types are representative of the main soil types in most smallholder farming communities in Zimbabwe and Southern Africa. As such, results have a broader applicability as inferential generalization can be done to other smallholder farming communities with similar settings.

To improve dependability of the study, a grounded theory approach to data analysis was adopted to enhance consistency of research findings with the data gathered. Results of the study were grounded in the narratives of smallholder farmers. In this regard, the probability of reproducing similar findings with similar subjects under similar circumstances was enhanced and improved. Confirmability was enhanced through detailed note taking, with audio recording complementing it. The notes were further used for reflective journalizing before transcription of farmers’ responses into textual data.

## 3. Results

### 3.1. Social impact of climate change on smallholder farmers

**Figure 1:**
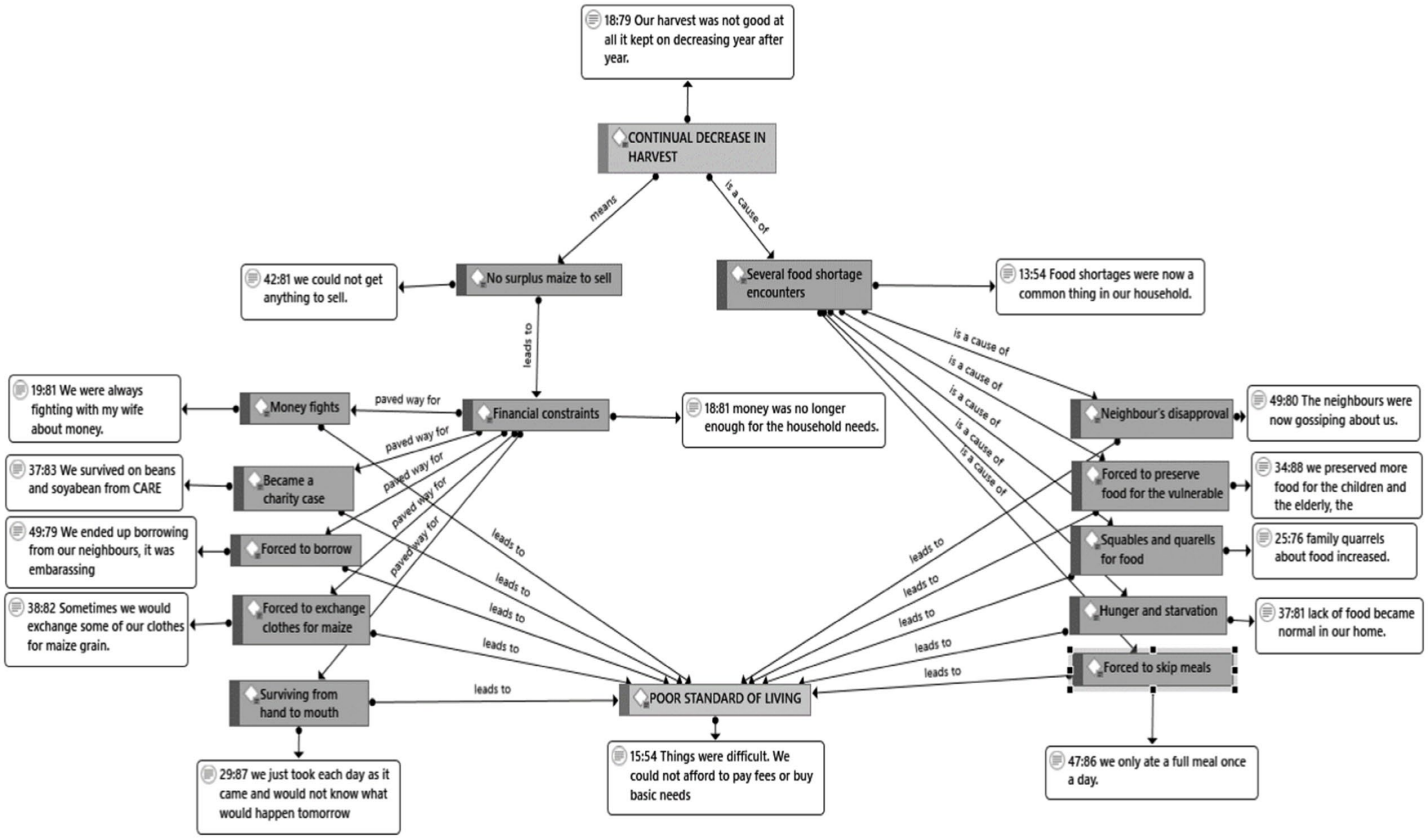
Social impacts of climate change on smallholder farmers.

Farmers experienced several social impacts due to climate change. Figure 1 is a network diagram generated from inductive coding and was imported from Atlas.ti. It shows the established codes (shaded) from the textual responses. The codes illustrate the social impact of climate change on farmers. Accompanying the network diagram are selected verbatim quotes (unshaded) that illustrate the findings. Continual decrease in yield caused a ripple effect of social impacts among smallholder farmers. Some farmers experienced food shortages and were forced to skip meals. Food shortages caused tensions and animosity among children. Farmers often reserved food for the vulnerable, in particular children, the elderly and those who were sick. There was no surplus to sell due to continuous decrease in yields. As a result, financial constraints were experienced resulting in money-induced conflicts among married couples. Some depended on support from donor organizations and borrowing to meet survival needs. Some farmers exchanged clothes and property for food. Other farmers lived on a hand to mouth basis with an uncertain future. All these social climate change impacts perpetuated and entrenched poverty and substandard standards of living among smallholder farmers.

**Table 1:** Psychological impact of climate change on smallholder farmers.

Selected verbatim quotes (Raw data)	Codes	Code group
“It is boring. We work so hard but there was nothing to show for it,” *Farmer 4, Ward 11*	Frustration	Negative
“We could not figure out what to do” *Farmer 48, Ward 20*	Hopeless	
“We did not know want else to do. It was defeat after defeat” *Farmer 21, Ward 12*	Defeated	
“I regretted coming to the resettlement” *Farmer 36, Ward 15*	Regret	
“No matter how much we tried, nothing worked out” *Farmer 22, Ward 12*	Futile efforts	
“I felt that it was my fault not being able to provide for my family” *Farmer 37, Ward 15*	Guilt	
“Going to the field is no longer interesting. We fail to harvest every year.” *Farmer 38, Ward 15*	Discouraged	
“We are always working in other people’s fields to get something to survive on” *Farmer 54, Ward 20*	Unbearable	
“I had failed as the head of the family” *Farmer 49, Ward 20*	Shame	
“We were always worried about what we will eat the following day” *Farmer 17, Ward 12*	Worry	Uncontrollable
“I was afraid. Looking at my children and how young they were” *Farmer 54, Ward 20*	Afraid	
“It was embarrassing to borrow food grain from our Neighbors” *Farmer 35, Ward 15*	Embarrassed	
“The variations persisted. We always think how the next season will be like” *Farmer 46, Ward 20*	Intimidated	
“The world is coming to an end. God is angry and is punishing us” *Farmer 9, Ward 11*	Devine punishment	Passive
“I did not know what else to do to sustain my family” *Farmer 13, Ward 11*	Lost	
“I thought, if only my husband was alive he would know what to do” *Farmer 18, Ward 12*	Bereavement	
“I thought, if we do not do something we were going to live in poverty” *Farmer 53, Ward 20*	Poverty prospects	
“It was painful to watch the crops wilt and die” *Farmer 24, Ward 12*	Hurt	
“I felt useless, there was nothing I could do” *Farmer 25, Ward 12*	Worthlessness	
“I had failed in every way to provide for my family” *Farmer 14, ward 11*	Failure	
“We needed a solution fast otherwise it was going to be a disaster” *Farmer 45, Ward 20*	Desperation	
“I thought of selling the plot and relocate to Harare to start something there” *Farmer 37, Ward 15*	Resigned acceptance	
“I was not happy. The situation was not looking good at all” *Farmer 28, Ward 12*	Sadness	
“I could see that if we do not do something we were going to die of hunger” *Farmer 3, Ward 11*	Coming to terms	Forceful
“We were not happy. We were angry at each other because there was no food” *Farmer 28, Ward 12*	Anger	
“I thought of selling the plot and relocate to Gokwe” *Farmer 43, Ward 20*	Abandoning	

### 3.2. Psychological impact of climate change on smallholder farmers

The social impacts listed above generated the psychological impacts among A1 farmers. Due to the several social impacts that farmers experienced, various feelings, thoughts, and emotions were encountered. The psychological impact of climate change originates from smallholder farmers’ feelings, thoughts and emotions about the impact caused by climate change. The psychological impacts of climate change on smallholder farmers is more extensive than the damage to things people care about. Table 1 shows verbatim quotes, established codes and associated code groups relating to the psychological impact of climate change on farmers. Psychological impacts of climate change display four main code groups.

**Table 2:** Implications of psychosocial impacts of climate change on sustainable development.

Impact	Specific impact	Implications on sustainable development	Potentially affected SDG	Specific SDG target potentially affected
Social	Food shortages; Hunger and starvation; Forced to preserve food; Squabbles and quarrels for food; Surviving from hand to mouth; Forced to skip meals; Forced to borrow; Became a charity case	Exacerbated hunger and starvation	SDG 2: Zero hunger	Target 2.1: End hunger and ensure access by all people to safe, nutritious and sufficient food
		Worsened food and nutrition insecurity		Target 2.2: End all forms of malnutrition and achieve targets on stunting and wasting in children under 5 years of age, address the nutritional needs of adolescent girls, pregnant and lactating women and elderly
	No surplus to sell; Financial constraints; Forced to exchange clothes for food	Farmers subjected to social and economic poverty	SDG 1: No poverty	Target 1.1: Eradicate extreme poverty for all people
	Money fights	Women exposed to domestic violence	SDG 5: Gender equality	Target 5.2: Eliminate all forms of violence against women
	Neighbor’s disapproval	Reduced social cohesion	SDG 13: Climate action	Target 13.1: Strengthen resilience and adaptive capacity to climate-related hazards and natural disasters in all countries
Psychological	Negative feelings	Threatens resilience building and increases exposure to climate related events among the poor	SDG 1: No poverty	Target 1.3: Build resilience of the poor in vulnerable situations and reduce exposure to climate related events
		Promotes maladaptive behavior	SDG 3: Good health and wellbeing	Target 3.d: Strengthen the capacity of all countries, in particular developing countries, for early warning, risk reduction and management of national and global health risks
	Uncontrollable feelings	Leads to maladaptive behavior, a barrier to taking action against climate impacts	SDG 13: Climate action	Target 13.3: Improve education, awareness-raising and human and institutional capacity on climate change mitigation, adaptation, impact reduction and early warning
	Passive feelings		SDG 2: Zero hunger	Target 2.3: Double agricultural productivity and incomes of small-scale food producers and family farmers
	Forceful feelings			

First, is the code group negative, which displays annoyance among farmers due to the inability to change circumstances in response to climate change. Despite working hard, A1 farmers’ efforts were said to be thwarted continuously. The farmers felt discouraged and regretted having moved to the resettlement areas. Others felt guilty and blamed themselves for the bad situation they found themselves in. The second code group was coined “uncontrollable feelings.” It comprised smallholder farmers who even though they knew the threat of climate change on farming operations, they could not do anything to combat it. All they could do was worry and remained uncertain about the future. Climate change intimidated farmers and they always wondered what the next season would be like.

The third code group was of those who had passive feelings. The farmers in this group demonstrated that they had accepted their situation and simply resigned to fate. For them, the circumstances that climate change brought before them could not be dealt with. It was interesting to find farmers giving a spiritual explanation as they highlighted that what they were experiencing was “divine punishment.” They indicated that God was angry and thus was punishing them for some misdeeds. They seemed to be preparing themselves for a life of shortages and were already coming to terms with their dire situation. In this category of farmers were widows who grieved in perpetuity, lamenting that the death of their loved ones marked the beginning of their suffering.

The fourth code group was that of farmers who exhibited forceful feelings because of unproductive solutions available to combat climate change shocks. They harbored thoughts of escaping the negative experiences by simply abandoning farming in the resettlement area. Among their thoughts was relocating to urban areas to seek employment. Others spoke of emigrating to Neighboring countries or embarking on illegal gold panning in disused mines.

### 3.3. Implications of psychosocial impact of climate change on sustainable development

To illustrate the implications of psychosocial impact of climate change, reference was made to the SDGs of the United Nations (2015). Table 2 summaries how the psychosocial impacts of climate change may affect the achievement of some of the SDGs and their targets. The affected SDGs are SDG 1 (No poverty), SDG 2 (Zero hunger), SDG 3 (Good health and wellbeing), SDG 5 (Gender equality), and SDG 13 (Climate Action).

Social impacts such as food shortages affect development initiatives through exacerbated hunger and starvation among marginalized farmers, worsening food, and nutrition security. Food shortages due to climate change pose challenges in achieving the SDG 2 targets 2.1 and 2.2 in emerging communities as few people will have less access to food and children are prone to malnutrition and other forms of diseases. Food shortages promote hunger due to lack of sufficient food required to meet a person’s daily requirements. Food shortages also exposes children to poor development, increased risk of infectious diseases, and stunted growth and are susceptible to chronic illnesses due to depressed immune systems as a result of insufficient and less nutritious food.

No surplus to sell due to reduced yields from climate change impacts results in financial constraints and smallholder farmers are subjected to both social and economic poverty. This has the potential to affect the achievement of SDG 1 specifically Target 1.1, which aims to eradicate extreme poverty by 2030. Climate change has the potential to propagate the vicious cycle of poverty among smallholder farmers in emerging rural communities. When smallholder farmers are subjected to poverty due to climate change impacts, they become more vulnerable and will be at more risk because they do not have the capacity to protect themselves. This makes it difficult for them to recover from climatic shocks as their main source of livelihoods is highly dependent on climate and they remain trapped in poverty. The female folk is exposed to domestic violence due to money fights that arise due to the financial constraints caused by the impact of climate change on smallholder farmer’s main source of livelihood. This can potentially affect achievement of SDG 5 specifically Target 5.2, which seeks to eliminate all forms of violence against women. Since the main source of livelihood could no longer sustain the family’s needs, smallholder farmers were confronted with financial pressures that could have triggered the money fights. Women are generally a vulnerable group when it comes to gender-based violence.

In some cases, smallholder farmers were ridiculed and gossiped about when they borrowed food from Neighbors. This shows reduced social cohesion. This affects SDG 13, in particular, Target 13.1, which aims to strengthen resilience and adaptive capacity to climate-related hazards and natural disasters in all countries. Reduced social cohesion has implications on adaptive capacity leading to reduced resilience. In most cases, disaster risk management requires that communities work together to achieve enhanced adaptive capacity and improved resilience. Reduced social cohesion could be a threat to achieving this target.

The psychological impacts have the potential to affect SGDs 1, 3, and 13. The negative feelings that the farmers had from the impact of climate change can threaten resilience building, have the potential to promote maladaptive behavior and could increase exposure of smallholder farmers to climate related impacts. Negative feelings among farmers tend to diminish efforts to act toward getting solutions to the climate change challenge. As such, efforts that may be directed toward combating climate change among farmers are likely to have low chances of being successful because smallholder farmers would be feeling discouraged. This has the potential to affect Target 1.3 of SDG 1 which aims to build resilience of the poor in vulnerable situations and reduce exposure to climate related events. Negative feelings also promote maladaptive behavior. Uncontrollable, passive and forceful feelings have the potential to deter smallholder farmers from taking action against climate change. This often lead to a number of negative consequences that increases risk and vulnerability thereby potentially affecting the achievement of SDGs 2 and 13, particularly Targets 2.3 and 13.3. Target 2.3 aims to double agricultural productivity and incomes of small-scale food producers and family farmers, however, if no action against climate change is taken it could be difficult to improve productivity. Target 13.3 aims to improve education, awareness-raising and human and institutional capacity on climate change mitigation, adaptation, impact reduction and early warning. However, if smallholder farmers mindsets are trapped in hopelessness there is a possibility that even if efforts are made toward improving education, awareness raising and capacity building would be fruitless.

## 4. Discussion

Psychosocial impacts of climate change manifest in various forms and are a potential threat to some of the SDGs and targets. The psychosocial impacts and associated outcomes are indirect, intangible and qualitative in nature. Presumably, this explains why they are overlooked in impact assessments. In addition, climate change is not seen as a social or psychological phenomenon with impacts beyond the biophysical (Doherty and Clayton, 2011). However, overlooking the psychosocial dimension would leave a gap in understanding the total impacts of climate change on farmers in rural communities.

Severe food shortages forced farmers to borrow. Borrowing is detestable and reproachful in societies (Baya et al., 2019). Thus, farmers suffered embarrassment and humiliation and sometimes subjected to ridicule and gossip from fellow community members. This could imply weak social cohesion among resettled farmers. Farmers seem unprepared to assist one another. Considering that the old resettlement was initiated in 1980 and the FTLRP in 2000, it is surprising that farmers are still not ready to assist each another. Social cohesion is an important pre-requisite for sustainable communities (Lieske et al., 2015). Hence, lack of or weakened social cohesion makes it impossible to achieve sustainable rural communities.

Reduction of number of meals per day although cited in many impact assessments, implications on dietary needs of family members, in particular children, the elderly and sick are not clear. Rarely is hunger considered (Hatcher et al., 2019) in climate change impact assessments. Neither are the consequences on daily nutritional requirements and overall health of vulnerable groups in society assessed. Farmers’ children squabbled and quarreled over food because it was not enough. This can be viewed within the context of “the social theory of survival of the fittest” (Darwin, 1859). Children who could fight and quarrel more, got the food. This would be unfair to weaker children. Although preserving food for the vulnerable is a plausible coping mechanism, it puts those who serve food in a precarious situation. Deciding on who to prioritize among the children, the elderly or sick relatives is a difficult task fraught with emotions that are difficult to manage.

Farmers regarded themselves as charity cases that depended on donor organizations. Such a mindset entrenches a dependency syndrome within the farming households (Chambers and Conway, 1992) leading to them being reluctant to improve their situation (Mudavanhu and Mandizvidza, 2013). Some farmers were of the belief that climate change impacts were impossible to deal with. These issues have severe consequences on sustainable development. Sustainable Development Goal 2 aims to end hunger and achieve food and nutrition security. However, climate change is exacerbating hunger and malnutrition while worsening food and nutrition insecurity among emerging rural communities.

Farmers exchanged clothes and other possessions for food. This observation confirms existing literature alluding to the fact that worsening food insecurity conditions leads to adoption of unsustainable means of survival (Harvey et al., 2014). As the farmers tried to balance food needs and cushion the impact of climate change, they resorted to unsustainable strategies (Torres-Vitolas et al., 2019). Exchanging clothes for food simply intensified the cycle of poverty. It is difficult to quantify the extent of unsustainability resulting from exchanging clothes for food because of the indirect and intangible nature of the social impact. As is the case with other qualitative variables, this has also been overlooked in climate impact assessments. Sustainable Development Goal 1 seeks to end all forms of poverty in all its forms. Nevertheless, climate change threatens these efforts as farmers are continually subjected to both social and economic poverty.

Money fights occurred due to financial constraints emanating from lack of surplus produce to sell. This illustrates strained interpersonal relationships. In general, men are dominant and acquire power through fulfilling gender-defined roles such as providing for families as stated in the Theoretical Masculinities Approach (Connell and Messerschmidt, 2005). Failure to fulfill the responsibilities, threatens their masculinity. Some get frustrated and become aggressive thus resorting to violence (Hatcher et al., 2019). Partner violence is a social issue that many communities face today. Sustainable Development Goal 5 aims to achieve gender equality and empower all women and girls with special focus on eliminating all forms of violence. Nonetheless, climate change impacts pose a threat to achieving this target.

Psychological impacts of climate change manifested in the form of farmers’ feelings, thoughts, and emotions about climate impacts. Most observations reflected cognitive and affective components of risk perception (Terpstra, 2011), all of which constitute a psychological dimension. The cognitive component focuses on farmers’ perceived risk of climate chnage impacts while the affective component comprised farmers’ feelings, thoughts and emotions toward climate change impacts.

Passive and uncontrollable feelings, thoughts and emotions of farmers can be perceived as low behavioral control (Gifford, 2011) and low self-efficacy (Grothmann and Patt, 2005) over climate change impacts. Both are psychological impacts that result in maladaptive behavior. Farmers believed they were incapable of carrying out adaptive responses. Although farmers were aware of the effect of climate change on farming as their main source of livelihood, they were not aware of the causes, its extent and the necessary actions to take. They could not fully comprehend the dynamics associated with climate change. As such, it is possible that perceived low self-efficacy was a result of farmers’ limited understanding about the causes and extent of climate change as well as the actions to take to reduce its effects (Gifford, 2011). This is limited cognition, which is a psychological impact identified as a barrier to taking action against climate change (Gifford, 2011). Passive and uncontrollable feelings can potentially affect SDGs 2 and 13, which aim to end all forms of hunger and combat climate change and its impacts, respectively. This is because passive and uncontrollable feelings lead to maladaptive behavior consequently reducing adaptive capacity.

Some farmers harbored thoughts of abandoning and escaping from the resettlement farms indicating loss of place attachment. Hidalgo and Hernandez (2001) regard this as the affective bond and/or link. The affective bond and/or link between farmers and resettlement areas had become weaker (Williams and Patterson, 1999) due to climate change impacts. This is surprising and reflect conflicting interests given that farmers had decided to leave former communal lands to resettle in Chirumanzu. It is possible to argue that this situation reflects the complex nature of climate change, which psychologically challenged the farmers to the extent that they questioned their previous decision of relocating to resettlement areas.

Thoughts of “divine punishment” show that farmers had lost religious conviction because of climate change impacts. Their trust in God was tried and tested and believed that they were being punished. Reflective psychological processes of this nature bound to religious beliefs disrupt the process of resilience building against climate change and increases farmers’ exposure to the effects. These issues are a threat to SDG 13 whose target is to improve human capacity on climate adaptation.

Some studies have demonstrated an appreciation of the importance of worry and increased anxiety, for example, Miceli et al. (2008) as an impact of climate change. However, they do not show how worry complicates farming processes. This study provides an explanation of how worry complicate farming operations. As farmers worried about their future due to recurring climate changes, they remained uncertain about the forthcoming farming season. Such circumstances made it difficult to plan resulting in farmers taking each day as it came. These complications demean resilience building vital to sustainable development.

## 5. Conclusion

Psychosocial impacts of climate change rarely feature in existing literature. Moreover, the effect of psychosocial impacts on sustainable development is unclear. This study advances the argument that psychosocial impacts of climate change should be integrated into impacts assessments and development issues. Evidence is provided from smallholder resettlement farmers’ experiences. Farmers agonized due to climate change impacts. They felt humiliated and embarrassed over the detestable practices they adopted to address food shortages. Farmers experienced heightened negative feelings, thoughts and emotions to an extent that they lost place attachment and religious conviction. Psychosocial impacts of climate change have the potential to negatively affect achievement of SDGs 1, 2, 3, 5, and 13. This study advocates for the adoption of a holistic approach in climate impact assessments, taking into consideration psychosocial impacts that are intangible, indirect and difficult to measure in quantitative terms to yield comprehensive evaluations. Psychosocial impacts of climate change should also be considered in development initiatives to avoid negative implications toward achievement of specific development targets. It is recommended that national governments should consider developing support mechanisms such as training and awareness programs for smallholder farmers to better cope with the psychosocial impacts of climate change, otherwise, they would be a huge barrier to development of emerging rural communities. Apart from that, more extension services are required among smallholder farmers to equip them with necessary skills to adjust to climate change and reduce the psychosocial impacts in the long run. Collective action could be one of the possible ways to reduce the psychosocial impact of climate change on sustainable development of emerging rural communities. Peer to peer knowledge sharing groups could be an essential platform for sharing information about the causes, extent and possible actions to take against climate change. Formal or informal cooperatives aimed at providing support for each other in times of need are also essential. Neighbor and community support programs could also be another possible way that can be explored in a bid to reduce the potential threat of the psychosocial impact of climate change on sustainable development of emerging rural communities.

## Data availability statement

The raw data supporting the conclusions of this article will be made available by the authors, without undue reservation.

## Ethics statement

The studies involving human participants were reviewed and approved by University of Venda Research and Ethics Committee. The patients/participants provided their written informed consent to participate in this study.

## Author contributions

DSK: conceptualization, data collection, data analysis, and manuscript preparation and validation.

## Conflict of interest

The author declares that the research was conducted in the absence of any commercial or financial relationships that could be construed as a potential conflict of interest.

## Publisher’s note

All claims expressed in this article are solely those of the authors and do not necessarily represent those of their affiliated organizations, or those of the publisher, the editors and the reviewers. Any product that may be evaluated in this article, or claim that may be made by its manufacturer, is not guaranteed or endorsed by the publisher.
